# Subjective Workload Perception and Coping Strategies: A Cross-Sectional Study of Polish Nurses

**DOI:** 10.3390/healthcare14193134

**Published:** 2026-09-22

**Authors:** Mária Lehotská, Katarzyna Tomaszewska, Krystyna Kowalczuk, Jozef Babečka

**Affiliations:** 1 Department of Nursing, Faculty of Health Sciences, Catholic University in Ružomberok, 034 01 Ružomberok, Slovakia; 2 Faculty of Health Care, Bronisław Markiewicz State Academy of Applied Sciences, 37-500 Jarosław, Poland; katarzyna.tomaszewska.nzoz@gmail.com; 3 Department of Integrated Medical Care, Medical University of Białystok, 15-096 Białystok, Poland; krystyna.kowalczuk@umb.edu.pl; 4 Faculty of Health Sciences, Catholic University in Ružomberok, 034 01 Ružomberok, Slovakia; jozef.babecka@ku.sk

**Keywords:** subjective workload, perception, psychological workload, nurses, Meister Questionnaire, Brief-COPE, coping strategies, occupational stress

## Abstract

**Highlights:**

**What are the main findings?**

- Nurses’ responses indicated a level of psychological workload at which temporary changes in subjective well-being and/or work performance may regularly occur, with time pressure, high responsibility, nervousness, fatigue, and long-term work tolerability being particularly prominent.
- Greater use of emotional support was associated with lower psychological workload, whereas more frequent substance use, self-distraction, and religious coping were associated with higher workload after accounting for other coping strategies and sociodemographic and occupational characteristics.

**What are the implications of the main findings?**

- Our findings highlight the importance of considering psychological workload in research and occupational strategies concerning nurses’ working conditions and well-being.
- Coping should be considered within the broader organizational and occupational context of psychological workload, while future research integrating subjective and objective workload measures may help inform more comprehensive strategies.

**Abstract:**

**Background/Objectives**: Subjective workload perception is an important occupational factor associated with nurses’ well-being, job satisfaction, and the quality of healthcare delivery. This study aimed to assess subjective workload perception in Polish nurses, identify their preferred coping strategies, and examine the associations between subjective workload perception, coping strategies, and selected sociodemographic and occupational characteristics. **Methods**: This cross-sectional study included 514 Polish nurses working in inpatient and outpatient healthcare settings. Data were collected using an anonymous online questionnaire comprising the Meister Questionnaire, the Brief-COPE Inventory, and items on sociodemographic and occupational characteristics. The 14 original Brief-COPE subscales were analyzed individually and additionally grouped for complementary analyses into problem-focused coping (active coping, planning, and instrumental support), emotion-focused coping (acceptance, emotional support, humor, positive reframing, and religion), and dysfunctional coping (behavioral disengagement, denial, self-distraction, self-blame, substance use, and venting). Group differences and associations were examined using non-parametric tests, Spearman’s rank correlations with Holm correction, and multivariable linear regression adjusted for sociodemographic and occupational characteristics. **Results**: Nurses’ responses indicated a level of psychological workload at which temporary changes in subjective well-being and/or work performance may regularly occur. Time pressure, high responsibility, nervousness, fatigue, and long-term work tolerability were particularly prominent aspects of psychological workload. Inferential comparisons of individual psychological workload scores showed no statistically significant between-group differences across the examined sociodemographic or occupational characteristics. Among individual coping strategies, emotional support showed the strongest inverse association with psychological workload (B = −1.73, 95% CI: −2.40 to −1.06, *p* < 0.001), whereas substance use showed the strongest positive association (B = 1.01, 95% CI: 0.46 to 1.55, *p* < 0.001). In complementary analyses, problem-focused coping was associated with lower psychological workload (B = −0.55, 95% CI: −0.78 to −0.32, *p* < 0.001), whereas dysfunctional coping was associated with higher workload (B = 0.57, 95% CI: 0.45 to 0.69, *p* < 0.001). **Conclusions**: The findings indicate that coping should be considered in relation to perceived psychological workload; however, the cross-sectional design precludes conclusions regarding causality.

## 1. Introduction

Nursing workload is an important occupational factor associated with nurses’ psychological well-being, job satisfaction, and the quality of healthcare delivery. The nursing profession involves substantial physical, psychological, and emotional demands. In addition to providing professional patient care, nurses are routinely exposed to intensive social interactions, time pressure, a high level of responsibility, and the need to make rapid decisions, which may contribute to their subjective perception of workload [1].

Traditionally, nursing workload has been defined as the volume of nursing activities that a nurse is required to perform within a given period of time [2]. Objective indicators, such as the number of patients, hours worked, or procedures performed, may not fully capture individual differences in perceived workload. A broader conceptualization was proposed by Alghamdi [3], who defined nursing workload as the amount of time and care nurses devote, directly and indirectly, to patients, the workplace, and professional development.

Contemporary approaches increasingly recognize workload as a multidimensional phenomenon that encompasses not only objective work demands but also their subjective perception. Subjectively perceived workload has been associated with occupational stress, burnout, job satisfaction, and psychological well-being among healthcare professionals [3,4].

High nursing workload has been associated with adverse outcomes for nurses and patients, with broader implications for healthcare systems. In a recent study among Greek nurses, higher workload was independently associated with higher burnout and turnover intention after adjustment for relevant occupational and sociodemographic characteristics [5]. Evidence regarding patient outcomes is also supported by a systematic review of intensive care settings, in which higher nursing workload was associated with increased mortality and adverse events [6]. Together, these findings highlight the relevance of nursing workload to both workforce well-being and the quality and safety of healthcare.

Current workforce trends provide an important context for understanding nursing workload. Persistent workforce shortages and increasing healthcare demands may increase the demands experienced by the available nursing workforce. These challenges are particularly relevant in Poland, which has 5.9 practicing nurses per 1000 population compared with an OECD average of 9.2, while current training rates remain insufficient to close this staffing gap [7,8].

Coping involves cognitive and behavioral efforts used to manage demands appraised as taxing or exceeding available resources [9]. In nursing, coping strategies have been associated with psychological well-being, perceived stress, burnout, and psychological distress [10,11,12]. Within the transactional model of stress and coping, individuals appraise environmental demands in relation to the resources available to manage them [9]. Considered alongside the distinction between objective work demands and their subjective perception described above, this framework provides a conceptual basis for examining associations between coping and psychological workload. Because coping strategies represent different ways of responding to demands appraised as stressful, the coping responses individuals employ may be associated with how they perceive psychological workload. Empirical findings among nurses further support this link: recent studies using the NASA-TLX have reported inverse associations between positive coping styles and mental workload, while a positive association between negative coping and mental workload has also been reported [13,14]. The direction of these relationships cannot, however, be assumed, as perceived workload may also be associated with the coping responses nurses employ.

Although these findings support an association between coping and mental workload, they provide limited insight into how this relationship is expressed at the level of specific coping responses and in relation to subjectively perceived psychological workload. Poku et al. [15], for example, assessed mental workload using the NASA-TLX alongside individual Brief-COPE strategies among nurses in Ghana. However, evidence on the associations between specific Brief-COPE strategies and subjectively perceived psychological workload remains limited. In particular, it remains unclear whether individual Brief-COPE strategies are independently associated with psychological workload when the strategies are considered simultaneously and sociodemographic and occupational characteristics are taken into account. Examining the 14 original Brief-COPE strategies as the primary analytical level, with broader coping dimensions providing a complementary perspective, may therefore offer a more differentiated understanding of the workload–coping relationship. Evidence from Central and Eastern European nursing populations also remains limited, providing an additional rationale for examining whether these associations are observed among Polish nurses when psychological workload and coping are assessed using instruments adapted for the target population. A better understanding of these associations may help inform future research and strategies addressing nurses’ psychological workload within its broader occupational and organizational context.

### Aim of the Study

The aim of this study was to assess the subjectively perceived psychological workload of Polish nurses using the Meister Questionnaire, identify preferred coping strategies using the Brief-COPE Inventory, and analyze the relationships between psychological workload, coping strategies, and selected sociodemographic and occupational characteristics.

## 2. Materials and Methods

This study employed a quantitative cross-sectional design. Data were collected between December 2024 and November 2025 using an anonymous online questionnaire. The questionnaire link was distributed electronically to nursing staff through deputy directors/directors of nursing at 15 hospitals located across the Polish voivodeships. Participants were recruited using a non-probability convenience sampling strategy with voluntary self-selection. Because distribution to individual nurses was managed locally by the participating hospitals, the exact number of nurses who received the questionnaire link was not recorded; consequently, a conventional response rate could not be calculated. A total of 514 complete questionnaires meeting the eligibility criteria were included in the final analysis.

Participation in the study was voluntary and anonymous. The inclusion criteria were as follows: (1) being a registered nurse according to the applicable legislation of the Republic of Poland; (2) being actively engaged in nursing practice at the time of data collection; (3) working in either inpatient or outpatient healthcare settings; (4) providing voluntary informed consent to participate in the study; and (5) completing the questionnaire in its entirety. Participants were excluded if they: (1) were not employed as nurses; (2) were nursing students without professional nursing practice; or (3) submitted an incomplete questionnaire.

No formal a priori sample-size calculation was performed before recruitment. To evaluate whether the achieved sample was adequate for the revised multivariable analyses, sample-size requirements were subsequently assessed for the largest regression model. Assuming α = 0.05, 80% power, 26 predictor degrees of freedom, and a medium overall effect size (Cohen’s f^2^ = 0.15), approximately 175 participants would be required. The final analytical sample comprised 514 participants.

The questionnaire consisted of three parts. The first part collected participants’ demographic and occupational characteristics, including sex, age, education, years of professional nursing practice, shift pattern, and type of care. The second part consisted of the Meister Questionnaire, which assesses subjectively perceived psychological workload. The questionnaire comprises 10 items addressing different aspects of psychological workload (e.g., “I often feel under time pressure at work”, “My work places a considerable psychological burden on me because of the high responsibility associated with serious consequences”, “My work is so psychologically exhausting that it cannot be performed for years with the same level of performance”). Each item is rated on a five-point Likert scale, where 5 = yes, I completely agree, 4 = somewhat yes, 3 = neither agree nor disagree, 2 = tend not to agree, and 1 = disagree, not at all. The Polish version of the Meister Questionnaire demonstrated good internal consistency in a validation study among nurses (Cronbach’s α = 0.83) [16].

The Meister Questionnaire was scored according to the original methodology described by Hladký and Židková [17]. The 10 items form three factors: Factor I (Overload; items 1, 3, and 5), Factor II (Monotony; items 2, 4, and 6), and Factor III (Non-specific factor; items 7–10). The overall raw score (HS) represents the sum of all 10 items and ranges from 10 to 50. The original methodology provides several related procedures for group-level evaluation. Individual questionnaire items are evaluated using group median scores and the corresponding predefined critical median values. Classification of the overall psychological workload of the group is based on factor scores obtained by summing the median scores of the items assigned to each factor. Table 1 presents the corresponding classification criteria and score ranges for workload Levels 1–3.

According to the original methodology, Level 1 represents psychological workload for which adverse effects on health, subjective well-being, or performance are unlikely.

Level 2 represents psychological workload in which temporary changes in subjective well-being and/or performance may regularly occur, whereas Level 3 represents workload for which health risks cannot be excluded [17].

In addition, the original Meister Questionnaire methodology provides a group-level factor evaluation based on arithmetic mean scores. For this purpose, mean scores for Factors I–III and the overall raw score (HS) are compared with sex-specific critical values provided in the original methodology. These critical values were defined as the corresponding reference mean + 0.5 SD. This reference-value comparison is distinct from the median-based workload classification procedure described above. For inferential statistical analyses in the present study, an individual HS was calculated for each participant by summing all 10 Meister Questionnaire items. Individual HS values, rather than group-level mean or median scores, were used as the psychological workload outcome in between-group comparisons, correlation analyses, and multivariable regression models, thereby preserving inter-individual variability.

In the present study, the Meister Questionnaire demonstrated excellent internal consistency (Cronbach’s α = 0.905).

The third part of the questionnaire consisted of the Brief-COPE Inventory, which was used to assess the coping strategies adopted by nurses when dealing with stressful situations. The Brief-COPE Inventory is a shortened 28-item version of the original 60-item COPE Inventory developed by Carver in 1997. The Polish adaptation of the instrument was developed by Juczyński and Ogińska-Bulik [18]. The Brief-COPE is a self-report instrument comprising 28 items assessing 14 original two-item coping subscales: self-distraction, denial, substance use, behavioral disengagement, emotional support, venting, humor, acceptance, self-blame, religion, active coping, instrumental support, positive reframing, and planning. Each coping strategy is assessed using two items. Participants responded to items addressing different ways of coping with stressful situations (e.g., “I turn to work or other activities to take my mind off things”, “I use alcohol or other substances to make myself feel better”, and “I take action to try to make the situation better”) on a four-point scale ranging from 0 (I almost never do this) to 3 (I almost always do this), where 1 = I rarely do this and 2 *=* I often do this. Each original two-item subscale therefore has a possible score range of 0–6. Scores for the 14 original Brief-COPE subscales were calculated by summing the two corresponding items according to the original Brief-COPE scoring: self-distraction (items 1 and 19), active coping (2 and 7), denial (3 and 8), substance use (4 and 11), use of emotional support (5 and 15), behavioral disengagement (6 and 16), venting (9 and 21), use of instrumental support (10 and 23), positive reframing (12 and 17), self-blame (13 and 26), planning (14 and 25), humor (18 and 28), acceptance (20 and 24), and religion (22 and 27).

The Brief-COPE was originally developed as an instrument comprising 14 separate subscales, whereas its higher-order factorial structure has remained inconsistent across subsequent studies. A systematic review by Solberg et al. [19] identified substantial variability in reported Brief-COPE factor structures, with solutions ranging from 2 to 15 factors. This variability has also been observed in nursing populations; for example, Halcomb et al. [20] identified a seven-factor solution among Australian primary health care nurses. Given this variability, the 14 original subscales were retained for the primary strategy-level analyses. For complementary analyses of broader coping patterns, the original subscales were additionally aggregated according to the three composite dimensions described and psychometrically evaluated by Cooper et al. [21]: problem-focused coping, emotion-focused coping, and dysfunctional coping.

Cooper et al. reported Cronbach’s α values of 0.84 for problem-focused coping, 0.72 for emotion-focused coping, and 0.75 for dysfunctional coping, together with evidence of temporal stability and validity. More recently, the three comparable Brief-COPE composite dimensions demonstrated high internal consistency in a sample of intensive and progressive care nurses, with Cronbach’s α values of 0.89 for problem-focused coping, 0.83 for emotion-focused coping, and 0.83 for the third composite dimension, termed dysfunctional/avoidant coping in that study [12]. This terminology reflects variation in the labeling of higher-order Brief-COPE dimensions across studies. A broader three-category grouping of Mini-COPE strategies into problem-focused, emotion-focused, and avoidant/dysfunctional coping has also been described by Cybulska et al. in a recent study among Polish nurses, although their analyses were conducted at the level of the 14 individual coping strategies rather than by empirically testing the three-dimensional structure [22]. Accordingly, the specific Cooper et al. dimensions were used in the present study as a complementary analytical framework rather than assumed to represent a universally established higher-order factorial structure of the Brief-COPE. According to the Cooper et al. framework [21], problem-focused coping comprised active coping, use of instrumental support, and planning (6 items; possible range 0–18). Emotion-focused coping comprised acceptance, use of emotional support, humor, positive reframing, and religion (10 items; possible range 0–30). Dysfunctional coping comprised behavioral disengagement, denial, self-distraction, self-blame, substance use, and venting (12 items; possible range 0–36). The composition of the three composite dimensions and the corresponding Brief-COPE subscales and item numbers are presented in Table 2.

Composite scores were calculated by summing the items assigned to the respective Cooper et al. dimensions. Because the three composite dimensions contained different numbers of items, standardized mean scores were additionally calculated by dividing each composite total score by the number of contributing items. This yielded scores on the original 0–3 response metric and enabled descriptive comparison of the relative frequency of use across the three dimensions.

For the Polish Mini-COPE adaptation, reliability coefficients for the individual subscales have been reported to range from 0.62 to 0.89 [18]. Cybulska et al. [22] reported Cronbach’s α coefficients ranging from 0.65 to 0.88 across the 14 subscales in a sample of Polish nurses. In the present study, internal consistency was evaluated for both the 14 original two-item subscales and the three composite dimensions. Because each original subscale comprises only two items and Cronbach’s α is sensitive to the number of items, inter-item correlations were additionally examined for the original subscales. Cronbach’s α for the 14 original subscales ranged from 0.460 to 0.874, with inter-item correlations ranging from 0.299 to 0.776. For the composite dimensions, Cronbach’s α was 0.811 for problem-focused coping, 0.758 for emotion-focused coping, and 0.836 for dysfunctional coping.

### 2.1. Statistical Analysis

Descriptive statistics included absolute and relative frequencies, means, medians, and standard deviations. Both instruments were scored in accordance with their respective scoring methodologies. Differences in the raw scores (HS) of the Meister Questionnaire across participant groups were assessed using non-parametric statistical tests because the analyzed variable did not meet the assumption of normality. For inferential analyses, categories with very small numbers of participants were combined on a conceptually meaningful basis. Age was analyzed in four categories (<30, 31–40, 41–50, and ≥51 years), education in three categories (secondary, Bachelor’s, and Master’s/Doctoral degree), years of nursing practice in five categories (<5, 5–10, 11–20, 21–30, and ≥31 years), and shift pattern as single-shift or multi-shift work. The original categories were retained for descriptive presentation of the study sample.

The Mann–Whitney U test was used for comparisons between two independent groups (sex, shift pattern, and type of care). For variables comprising three or more independent groups (age group, educational attainment, and years of nursing practice), the Kruskal–Wallis test was applied as a non-parametric alternative to one-way analysis of variance (ANOVA). Effect sizes were reported as r for Mann–Whitney U tests and epsilon-squared (ε^2^) for Kruskal–Wallis tests.

The relationships between coping strategies assessed using the Brief-COPE Inventory and individual Meister HS values were examined using Spearman’s rank correlation coefficient (ρ). Correlation analyses were performed for both the 14 original Brief-COPE subscales and the three composite coping dimensions. To account for multiple testing, Holm’s correction was applied separately to the 14 original subscales and to the three composite dimensions. Both unadjusted and Holm-adjusted *p*-values were reported.

Two multivariable linear regression models were fitted with individual Meister HS as the dependent variable. The primary strategy-level model included all 14 original Brief-COPE subscales simultaneously, together with sex, age group, education, years of nursing practice, shift pattern, and type of care as covariates. The complementary composite-level model included the three composite dimensions—problem-focused coping, emotion-focused coping, and dysfunctional coping—instead of the 14 individual subscales, while retaining the same covariates.

Categorical predictors were dummy-coded using female sex, age < 30 years, secondary education, <5 years of nursing practice, single-shift work, and inpatient care as reference categories. Regression results were reported as unstandardized coefficients (B), standard errors (SE), standardized coefficients (β), 95% confidence intervals (CI), and *p*-values. For categorical predictors with more than two levels, overall tests were additionally performed. Model fit was summarized using R^2^, adjusted R^2^, and the overall F-test.

Regression assumptions were evaluated using residual diagnostics. Multicollinearity was assessed using variance inflation factors (VIF), and heteroskedasticity was assessed using the Breusch–Pagan test. Because residual normality was not fully satisfied, HC3 heteroskedasticity-robust standard errors were additionally examined in sensitivity analyses to assess the robustness of the regression findings.

Statistical analyses were performed using IBM SPSS Statistics for Windows, Version 28.0 (IBM Corp., Armonk, NY, USA).

Statistical significance was established at α = 0.05, and *p*-values < 0.05 were considered statistically significant.

### 2.2. Ethical Considerations

The study was conducted in accordance with the principles of the Declaration of Helsinki. Ethical approval was obtained from the Bioethical Committee of PWSW in Przemyśl (approval No. KBPANS/12/2024, dated 5 November 2024). Participation in the study was voluntary and anonymous. Before accessing the questionnaire, respondents were provided with information about the purpose of the study, anonymity, and the voluntary nature of participation. Completion of the anonymous online questionnaire was considered to indicate informed consent. No personally identifiable information was collected, and all data were analyzed in aggregate form to ensure confidentiality.

## 3. Results

### 3.1. Characteristics of the Study Sample

A total of 514 Polish nurses were included in the final analysis. Their sociodemographic and occupational characteristics are presented in Table 3.

### 3.2. Psychological Workload Assessed Using the Meister Questionnaire

In the study sample, the median value for five out of the ten scaled items of the Meister Questionnaire was equal to or higher than the critical median value for the respective item (Table 4).

As shown in Table 4, the median values for time pressure (Me = 4) and long-term work tolerability (Me = 3) exceeded the corresponding critical median values by 0.5–1 scale point. Median values equal to the corresponding critical median values were observed for the items assessing perceived nervousness, fatigue, and responsibility at work. In contrast, the critical median value was not reached for the remaining items.

The median-based factor scores were 9 points for Factor I (Overload), 6 points for Factor II (Monotony), and 11 points for Factor III (Non-specific factor), as presented in Table 5.

As Factor I exceeded Factor II by three points, thus meeting the ≥2-point criterion, the overload classification pathway was applied. The combined score of Factors I and III was 20 points, corresponding to Level 2 psychological workload with a tendency toward overload according to the Meister Questionnaire classification procedure.

According to the group-level factor evaluation, mean scores were compared with the sex-specific critical values provided in the original Meister Questionnaire methodology (Table 6). Mean factor scores remained below the corresponding critical values in both sexes. The mean HS was below the critical value among women and slightly above the corresponding critical value among men.

### 3.3. Differences in Psychological Workload According to Selected Sociodemographic and Occupational Characteristics

Inferential comparisons of individual Meister Questionnaire raw scores (HS) showed no statistically significant between-group differences according to sex, age group, education, years of nursing practice, shift pattern, or type of care (all *p* > 0.05). Effect sizes were negligible to small (Table 7).

### 3.4. Coping Strategies Assessed Using the Brief-COPE Inventory

Table 8 presents descriptive statistics for the 14 original Brief-COPE subscales and the three composite dimensions. At the strategy level, active coping showed the highest standardized mean score (1.89), followed by emotional support and planning (both 1.83) and instrumental support (1.76). Substance use showed the lowest standardized mean score (0.59), followed by behavioral disengagement (0.88) and humor (0.94). Among the composite dimensions, problem-focused coping had the highest standardized mean score (1.83), followed by emotion-focused coping (1.46) and dysfunctional coping (1.08).

### 3.5. Associations Between Psychological Workload and Coping Strategies

Spearman’s correlation analysis showed statistically significant associations between psychological workload and 12 of the 14 original Brief-COPE subscales, all of which remained significant after Holm correction for multiple testing (Table 9). Positive associations were observed for substance use (ρ = 0.349), behavioral disengagement (ρ = 0.300), denial (ρ = 0.255), self-blame (ρ = 0.245), humor, self-distraction, venting, and religion. Inverse associations were observed for emotional support (ρ = −0.294), planning (ρ = −0.233), instrumental support (ρ = −0.169), and active coping (ρ = −0.148). Positive reframing and acceptance were not significantly associated with psychological workload after Holm correction.

At the composite level, dysfunctional coping was positively associated with psychological workload (ρ = 0.356), whereas problem-focused coping was inversely associated with psychological workload (ρ = −0.224); both associations remained statistically significant after Holm correction. Emotion-focused coping was not significantly associated with psychological workload (ρ = −0.018).

To further examine these associations while accounting for sociodemographic and occupational characteristics, multivariable linear regression analyses were performed (Table 10).

In the primary strategy-level multivariable model, which included all 14 original Brief-COPE subscales and sociodemographic and occupational covariates, emotional support was independently associated with lower psychological workload (B = −1.728, 95% CI: −2.395 to −1.061, *p* < 0.001). Substance use (B = 1.006, 95% CI: 0.461 to 1.552, *p* < 0.001), self-distraction (B = 0.740, 95% CI: 0.170 to 1.309, *p* = 0.011), and religion (B = 0.510, 95% CI: 0.096 to 0.924, *p* = 0.016) were independently associated with higher psychological workload. The remaining original Brief-COPE subscales were not statistically significant in the adjusted model. None of the sociodemographic or occupational covariates showed a statistically significant independent association with psychological workload. The model explained 28.3% of the variance in individual Meister Questionnaire raw scores (adjusted R^2^ = 0.245; F(26, 487) = 7.40, *p* < 0.001) (Table 10).

In the complementary composite-level model, problem-focused coping was independently associated with lower psychological workload (B = −0.553, 95% CI: −0.785 to −0.322, *p* < 0.001), whereas dysfunctional coping was independently associated with higher psychological workload (B = 0.569, 95% CI: 0.447 to 0.692, *p* < 0.001). Emotion-focused coping was not statistically significantly associated with psychological workload (B = −0.064, 95% CI: −0.254 to 0.127, *p* = 0.513). None of the sociodemographic or occupational covariates were statistically significant. The model explained 22.6% of the variance in individual Meister Questionnaire raw scores (adjusted R^2^ = 0.203; F(15, 498) = 9.69, *p* < 0.001) (Table 10).

## 4. Discussion

The aim of this study was to assess the subjectively perceived psychological workload of Polish nurses, identify their preferred coping strategies, and examine the relationships between psychological workload, coping strategies, and selected sociodemographic and occupational characteristics. According to the Meister Questionnaire methodology, the study sample demonstrated Level 2 psychological workload with a tendency toward overload, with time pressure, high responsibility, nervousness, fatigue, and long-term work tolerability reaching or exceeding their corresponding critical median values. Inferential comparisons of individual Meister Questionnaire raw scores (HS) showed no statistically significant between-group differences across the examined sociodemographic and occupational characteristics. In the multivariable strategy-level model, emotional support was independently associated with lower psychological workload, whereas substance use, self-distraction, and religion were independently associated with higher psychological workload. At the composite level, problem-focused coping was independently associated with lower psychological workload, whereas dysfunctional coping showed an independent positive association with psychological workload. These findings highlight differences in the associations of individual coping strategies and broader coping dimensions with psychological workload.

According to the Meister Questionnaire methodology, the study sample was classified as Level 2 psychological workload with a tendency toward overload, whereas the arithmetic mean scores of the individual workload factors remained below the corresponding population reference critical values. These apparently differing findings are not contradictory but reflect the two complementary group-level evaluation procedures described in the original methodology.

Comparison of our findings was based primarily on studies that employed the Meister Questionnaire to assess psychological workload. As this instrument has been used predominantly in Central European countries, particularly the Czech Republic, Slovakia, and, to a lesser extent, Poland, most comparable studies originate from this region. Similar findings were reported by Obrončíková et al. [23], who investigated psychological workload among Slovak nurses working in neurological wards and intensive care units. According to the Meister methodology, nurses working in general wards demonstrated Level 1 workload, whereas nurses employed in intensive care units reached Level 2 workload. However, comparisons of mean raw scores and factor scores with population reference values did not exceed the corresponding critical values in either group. Similar to the present study, several of the more prominent workload indicators were related to time pressure, responsibility, fatigue, nervousness, and long-term work tolerability. Consistent with the broader conceptualization proposed by Alghamdi [3], nursing workload should be considered in relation to factors associated with patients, nurses, and the healthcare organization. Our findings are consistent with those reported by Dimunová et al. [24], who likewise identified Level 2 psychological workload among intensive care nurses, with the critical value exceeded for Factor I (Overload). In their study, the critical median values were exceeded particularly for time pressure and long-term work tolerability. Using a different multidimensional approach to workload assessment, Umansky and Rantanen [25] also identified time pressure as one of the factors contributing to nurses’ workload. The broader relevance of time pressure in nursing is also supported by findings from the European Nurses’ Early Exit Study, in which time pressure and concerns regarding quality of care were among the most frequently reported work-related factors contributing to nurses’ decisions to leave their institutions [26].

A systematic review and meta-analysis by Yuan et al. [27], including 31 studies and 16,189 nurses, reported a pooled prevalence of high mental workload of 54%. Although the included studies assessed mental workload using the NASA-TLX rather than the Meister Questionnaire, the findings indicate that high mental workload is common across diverse nursing settings.

Time pressure has also been identified as a key component of nursing workload in a qualitative systematic review, which highlighted time constraints, inadequate staffing, and limited resources as important contributors to heavy nursing workload [28]. The findings of the present study suggest that perceived time pressure may reflect the time demands associated with nursing care. However, examining this possibility requires the integration of subjective assessments with objective workload measures, such as analyses of the time devoted to individual nursing interventions using the Nursing Interventions Classification (NIC).

Inferential comparisons of individual Meister Questionnaire raw scores (HS) showed no statistically significant between-group differences according to sex, age, educational attainment, years of nursing practice, shift pattern, or type of care. This result should be distinguished from the descriptive comparison of mean HS values with sex-specific population reference values, in which the mean HS remained below the critical value among women but was slightly above the corresponding critical value among men. The two approaches address different questions: the inferential analysis evaluates between-group differences in individual HS values, whereas the reference-value comparison evaluates how a group mean relates to an external sex-specific threshold. Accordingly, the slight exceedance of the reference value among men should not be interpreted as evidence of higher psychological workload in men than in women. The inferential pattern was also reflected in the multivariable analyses, in which none of the sociodemographic or occupational covariates showed a statistically significant independent association with psychological workload. Overall, the examined sociodemographic and occupational characteristics may have limited ability to distinguish differences in subjectively perceived psychological workload within the present sample. Recent systematic evidence among intensive care unit nurses identified demographic, work-related, and psychological factors as potential influences on mental workload, supporting its multifactorial nature [29].

The absence of a statistically significant between-group difference in individual HS values according to type of care is consistent with our previous study published in 2025, which compared subjectively perceived psychological workload among outpatient and inpatient nurses in Slovakia. No substantial differences were observed in either the level or tendency of psychological workload, and analyses of both individual questionnaire items and Meister Questionnaire factor scores revealed only minimal differences between the two groups [30]. Similarly, Hozová and Vrublová [31] reported no significant differences in perceived workload according to workplace assignment, hospital department, or job position among non-medical healthcare professionals under routine working conditions. Direct comparisons of psychological workload between nurses working in outpatient and inpatient settings remain relatively limited, and further research is needed to determine whether workload differs systematically between these settings.

Our findings regarding age differ from those reported by Dębska et al. [32], who found that age was significantly associated with selected dimensions of psychological workload assessed using the Meister Questionnaire. Differences in sample composition, categorization of age groups, and analytical approaches may contribute to the differing findings. In the present study, categories with very small numbers of participants were combined for inferential analyses to improve the robustness of the comparisons.

At the strategy level, active coping, planning, emotional support, and instrumental support were among the most frequently used coping strategies, whereas substance use, behavioral disengagement, and humor were among the least frequently used. Similar patterns have been reported among Polish nurses [33,34].

Although the emotion-focused coping composite dimension had a lower standardized mean score than the problem-focused coping composite dimension, some of its individual strategies, particularly emotional support and acceptance, were among the more frequently used coping strategies. This pattern highlights the heterogeneity of individual coping strategies within broader composite dimensions and supports their interpretation alongside the 14 original Brief-COPE subscales. A similar strategy-level approach was adopted by Barr [35], who examined the 14 Brief-COPE coping styles individually rather than aggregating them into broader dimensions.

At the bivariate level, significant associations with psychological workload were observed for 12 of the 14 individual Brief-COPE strategies, all of which remained significant after Holm correction for multiple testing. However, after simultaneous adjustment for the other Brief-COPE subscales and sociodemographic and occupational characteristics, only emotional support, substance use, self-distraction, and religion remained independently associated with psychological workload. Emotional support was associated with lower psychological workload, whereas substance use, self-distraction, and religion were associated with higher psychological workload. The remaining bivariate associations were no longer statistically significant in the adjusted model.

Emotional support showed the strongest independent inverse association with psychological workload among the individual coping strategies. It was also among the more frequently used coping strategies in the present study. Laus et al. [36] similarly reported inverse associations of emotional support with burnout exhaustion and disengagement among Italian nurses exposed to verbal workplace violence. Their study examined burnout rather than psychological workload; nevertheless, the direction of these associations is consistent with the inverse association observed for emotional support in the present study.

Substance use showed a contrasting pattern. It was the least frequently used coping strategy in the present sample, yet it showed the strongest positive bivariate association with psychological workload among the individual Brief-COPE subscales and remained independently associated with higher psychological workload in the adjusted strategy-level model. This finding indicates that the frequency with which a coping strategy is used should be distinguished from the strength of its association with psychological workload. An integrative review by Mercer et al. [37] identified occupational stress and trauma among the psychosocial factors associated with alcohol use in nurses and reported evidence of alcohol being used as a coping response to workplace stress. Given the cross-sectional design of the present study, the direction of the observed association between substance use and psychological workload cannot be determined.

Self-distraction and religion were also independently associated with higher psychological workload in the adjusted strategy-level model. A positive association involving religion was also reported by Laus et al. [36], who found that the Brief-COPE religion subscale was independently associated with higher burnout disengagement among Italian nurses exposed to verbal workplace violence (β = 0.30, *p* = 0.001). This association was not consistently observed across their regression models, suggesting that the relationship between religious coping and psychological outcomes may vary according to the outcome and occupational context. The positive association may also reflect greater reliance on religious coping among nurses experiencing higher psychological workload, rather than an adverse role of religious coping itself. The finding should therefore not be interpreted as indicating that religious coping is inherently maladaptive.

The complementary composite-level analysis supported the broader pattern observed at the strategy level, with problem-focused coping associated with lower psychological workload and dysfunctional coping with higher psychological workload, whereas emotion-focused coping was not significantly associated with workload. Importantly, the absence of an association for the emotion-focused composite contrasted with the inverse association observed for emotional support at the individual-strategy level, further highlighting the heterogeneity of strategies grouped within broader coping dimensions.

The inverse association observed for problem-focused coping is consistent with findings by Zeladita-Huaman et al. [11], who found that higher problem-focused coping was independently associated with lower perceived stress among Peruvian nurses (B = −0.15, *p* = 0.002). More direct evidence for a workload–coping relationship has recently been reported among clinical nurses. Jin et al. [13] reported an inverse association between positive coping and mental workload and a positive association between negative coping and mental workload, while Ren et al. [14] also reported an inverse association between positive coping and mental workload. Although these studies used the NASA-TLX and a different coping instrument, their findings provide direct comparative evidence of associations between coping patterns and nurses’ mental workload.

Recent findings by Hackney and Surgenor [12] provide additional context for the composite-level results. Using comparable Brief-COPE composite dimensions among intensive and progressive care nurses, they found that all three coping dimensions contributed significantly to models of emotional exhaustion and depersonalization after accounting for relevant covariates. Their findings therefore partly align with the present results but also illustrate differences across psychological outcomes. In particular, dysfunctional coping showed significant associations with burnout outcomes, consistent with the positive association between dysfunctional coping and psychological workload observed in the present study, whereas emotion-focused coping was also significant in their models but was not associated with psychological workload in our composite-level analysis.

The strategy-level model explained 28.3% of the variance in psychological workload, whereas the complementary composite-level model explained 22.6%. These findings indicate that coping strategies, together with the sociodemographic and occupational characteristics included in the models, accounted for only part of the variability in perceived psychological workload. A substantial proportion remained unexplained and may be related to factors not assessed in the present study, including characteristics of the work environment, objective workload, organizational and interpersonal factors, and other individual characteristics.

This interpretation is consistent with the transactional model of stress proposed by Lazarus and Folkman [9], in which coping represents one component of a broader process involving the appraisal of environmental demands and available resources. From this perspective, psychological workload is unlikely to be explained by coping strategies alone but rather reflects the interplay between individual responses and the demands and resources of the work environment.

From this perspective, the present findings support considering coping within the broader organizational and occupational context of psychological workload rather than as an isolated individual-level factor. Future longitudinal studies integrating subjective and objective workload measures are needed to clarify the direction of the observed associations and to determine whether changes in coping strategies are associated with subsequent changes in psychological workload.

### Strengths and Limitations

The findings of this study should be interpreted in light of several limitations. First, the cross-sectional design precludes conclusions regarding causal or directional relationships between subjectively perceived psychological workload and coping strategies. Second, participants were recruited using a convenience sampling strategy from healthcare facilities in Poland, which may limit the generalizability of the findings to the broader population of Polish nurses. Furthermore, all data were collected using self-report questionnaires and may therefore have been influenced by reporting bias, including social desirability bias and inaccuracies in self-assessment.

Another limitation concerns the distribution of participants across some sociodemographic and occupational categories. To avoid sparse groups in the inferential analyses, categories with small numbers of participants were combined into broader groups, as described in the statistical analysis. This approach improved the suitability of the between-group comparisons but reduced the granularity of the analyses and may have obscured differences between some of the original categories.

The interpretation of the composite Brief-COPE dimensions also warrants caution. The composite framework used in the complementary analyses has been psychometrically evaluated, and comparable composite dimensions have demonstrated satisfactory to high internal consistency in previous research; however, no universally accepted higher-order factor structure of the Brief-COPE has been established. For this reason, the 14 original Brief-COPE subscales were retained as the primary level of analysis, whereas the composite dimensions were used as a complementary analytical framework.

Finally, the present study evaluated only subjectively perceived psychological workload. Objective indicators of workload, such as the time required to perform nursing interventions, the number of nursing activities completed during a work shift, staffing levels, or patient workload, were not included in the analyses. The present study represents one phase of the ongoing Nursing Workload research project (No. 011KU-4/2024). Data on objective nursing workload, including time measurements of nursing interventions based on the Nursing Interventions Classification (NIC), are currently being collected and analyzed. Integrating subjective and objective workload measures in future analyses may provide a more comprehensive understanding of the factors associated with nurses’ psychological workload.

Despite these limitations, the study has several strengths. It included a relatively large sample of 514 Polish nurses employed across different healthcare settings. The use of two standardized instruments, the Meister Questionnaire and the Brief-COPE Inventory, enabled psychological workload and coping strategies to be assessed using established measurement approaches, with the reliability of the measures evaluated in the present sample. In addition, psychological workload was evaluated using both group-level procedures provided in the original Meister Questionnaire methodology: workload classification based on factor scores and comparison of arithmetic mean scores with population reference critical values. The statistical analysis further incorporated correction for multiple testing in the correlation analyses and multivariable regression models accounting simultaneously for coping strategies and sociodemographic and occupational characteristics. Together, these methodological features provide a comprehensive and analytically robust assessment of the relationships between coping strategies and subjectively perceived psychological workload.

## 5. Conclusions

According to the Meister Questionnaire methodology, the study sample demonstrated Level 2 psychological workload with a tendency toward overload, indicating a level at which temporary changes in subjective well-being and/or performance may regularly occur. Time pressure, high responsibility, nervousness, fatigue, and long-term work tolerability reached or exceeded their corresponding critical median values.

The findings indicate that coping is associated with perceived psychological workload, but that these relationships differ across individual coping strategies and broader coping dimensions. At the strategy level, emotional support showed the strongest inverse association with psychological workload, whereas substance use showed the strongest positive association. At the composite level, problem-focused coping was associated with lower psychological workload, whereas dysfunctional coping was associated with higher workload; emotion-focused coping showed no significant association. Together, these findings support considering individual coping strategies while using broader composite dimensions as a complementary perspective. Given the cross-sectional design, the observed associations should not be interpreted as causal. Future longitudinal research integrating subjective and objective workload measures may provide a more comprehensive understanding of the relationships between coping and nurses’ psychological workload.

## Figures and Tables

**Table 1:** Classification of psychological workload according to the Meister Questionnaire.

Workload Tendency	Score Range	Workload Level
Overload tendency (Factors I + III)	7–19	1
	20–24	2
	25–35	3
Monotony tendency (Factors II + III)	7–21	1
	22–26	2
	27–35	3
Non-specific workload (Factors I + II + III)	10–26	1
	27–32	2
	33–50	3

Note: According to Meister’s original methodology, an overload tendency is classified when Factor I exceeds Factor II by ≥2 points. A monotony tendency is classified when Factor II exceeds Factor I by ≥2 points. If the difference between Factors I and II is <2 points, the workload is classified as non-specific [17].

**Table 2:** Classification of coping dimensions.

Coping Dimension	Brief-COPE Domains	No. of Items	Possible Score
Problem-focused coping	Active coping (2, 7)	6	0–18
	Planning (14, 25)		
	Instrumental support (10, 23)		
Emotion-focused coping	Emotional support (5, 15)	10	0–30
	Positive reframing (12, 17)		
	Humor (18, 28)		
	Acceptance (20, 24)		
	Religion (22, 27)		
Dysfunctional coping	Self-distraction (1, 19)	12	0–36
	Denial (3, 8)		
	Substance use (4, 11)		
	Behavioral		
	disengagement (6, 16)		
	Venting (9, 21)		
	Self-blame (13, 26)		

**Table 3:** Characteristics of the study participants (*N* = 514).

Characteristic	Category	*n*	%
Sex	Female	468	91.05
	Male	46	8.95
Age group (years)	<30	267	52.00
	31–40	107	20.80
	41–50	83	16.10
	51–60	51	9.90
	≥61	6	1.20
Education	Secondary school	89	17.32
	Bachelor’s degree	261	50.78
	Master’s degree	155	30.16
	Doctoral degree	9	1.75
Years of nursing practice	<5	321	62.45
	5–10	53	10.31
	11–20	48	9.34
	21–30	60	11.67
	31–40	27	5.25
	>40	5	0.97
Shift pattern	Day shift only	213	41.44
	Two 12-h shifts	272	52.92
	Two- or three-shift schedule	17	3.31
	Three 8-h shifts	12	2.33
Type of care	Inpatient care	447	86.96
	Outpatient care	67	13.04

**Table 4:** Median scores for individual Meister Questionnaire items and corresponding critical values.

Questionnaire Item	Factor	Median	Critical Value
**Time pressure**	**I**	**4**	**3.0**
Low job satisfaction	II	2	2.5
**High responsibility**	**I**	**3**	**3.0**
Numbing work	II	2	2.5
Problems and conflicts	I	2	2.5
Monotony	II	2	2.5
**Nervousness**	**III**	**3**	**3.0**
Mental satiation	III	2	3.0
**Fatigue**	**III**	**3**	**3.0**
**Long-term work tolerability**	**III**	**3**	**2.5**

Note: Bold rows indicate questionnaire items for which the median score was equal to or exceeded the corresponding critical value defined in the Meister Questionnaire. Factor I = Overload; Factor II = Monotony; Factor III = Non-specific factor.

**Table 5:** Factor scores calculated from the Meister Questionnaire.

Factor No.	Factor Name	Questionnaire Items	Factor Scores
I	Overload	1 + 3 + 5	9
II	Monotony	2 + 4 + 6	6
III	Non-specific factor	7 + 8 + 9 + 10	11

Note: Factor scores represent the sum of the median values for the questionnaire items assigned to each Meister Questionnaire factor.

**Table 6:** Mean factor scores and comparison with population critical values.

Sex	Factor No.	Factor Name	Mean ± SD	Population Critical Value
Women	I	Overload	9.25 ± 2.78	10.00
	II	Monotony	6.51 ± 2.81	9
	III	Non-specific factor	10.29 ± 4.17	14
	HS	Overall raw score	26.05 ± 8.55	29
Men	I	Overload	9.02 ± 2.68	12
	II	Monotony	7.20 ± 2.99	8
	III	Non-specific factor	10.48 ± 4.02	12
	HS	Overall raw score	26.70 ± 8.52	26

Note: Sex-specific critical values were obtained from the original Meister Questionnaire methodology. Critical values were defined as the reference mean + 0.5 SD. HS = overall raw score.

**Table 7:** Association between psychological workload and respondent characteristics.

Variable	Groups	*n*	HS Mean ± SD	Test Statistic	*p*-Value	Effect Size
Sex	Female	468	26.05 ± 8.55	U = 9930.5	0.386	r = 0.077
	Male	46	26.70 ± 8.52			
Age group (years)	<30	267	26.43 ± 8.72	H = 1.95	0.582	ε^2^ = 0.000
	31–40	107	26.01 ± 8.37			
	41–50	83	24.86 ± 8.74			
	≥51	57	26.60 ± 7.71			
Education	Secondary school Bachelor’s degree Master’s/Doctoral degree	89	27.92 ± 9.49	H = 3.66	0.161	ε^2^ = 0.003
		261	25.47 ± 8.31			
		164	26.13 ± 8.27			
Years of nursing practice	<5	321	26.05 ± 8.42	H = 2.36	0.670	ε^2^ = 0.000
	5–10	53	25.91 ± 9.22			
	11–20	48	25.54 ± 9.14			
	21–30	60	27.20 ± 8.73			
	≥31	32	25.81 ± 7.63			
Shift pattern	Single-shift work Multi-shift work	213	26.05 ± 7.90	U = 32,128.0	0.966	r = 0.002
		301	26.15 ± 8.98			
Type of care	Inpatient care Outpatient care	447	26.11 ± 8.67	U = 15,076.0	0.929	r = 0.007
		67	26.04 ± 7.65			

Note: HS = individual overall raw score of the Meister Questionnaire; SD = standard deviation; U = Mann–Whitney U statistic; H = Kruskal–Wallis statistic; r = effect size for Mann–Whitney U tests; ε^2^ = epsilon-squared effect size for Kruskal–Wallis tests. For inferential analyses, categories with very small sample sizes were consolidated as described in the Statistical Analysis section.

**Table 8:** Overall use of coping strategies.

Original Brief-COPE Subscales	Possible Score	Mean Total Score ± SD	Standardized Mean	Cronbach’s α	Inter-Item r
Active coping	0–6	3.79 ± 1.39	1.89	0.580	0.409
Emotional support	0–6	3.66 ± 1.55	1.83	0.677	0.512
Instrumental support	0–6	3.51 ± 1.58	1.76	0.702	0.541
Positive reframing	0–6	3.33 ± 1.42	1.67	0.578	0.407
Planning	0–6	3.66 ± 1.47	1.83	0.683	0.519
Venting	0–6	2.63 ± 1.44	1.32	0.462	0.300
Humor	0–6	1.88 ± 1.49	0.94	0.494	0.328
Acceptance	0–6	3.27 ± 1.47	1.63	0.678	0.513
Self-blame	0–6	2.35 ± 1.65	1.18	0.727	0.571
Religion	0–6	2.46 ± 1.78	1.23	0.806	0.675
Self-distraction	0–6	3.10 ± 1.50	1.55	0.460	0.299
Substance use	0–6	1.18 ± 1.66	0.59	0.874	0.776
Denial	0–6	1.97 ± 1.45	0.98	0.605	0.434
Behavioral disengagement	0–6	1.76 ± 1.48	0.88	0.671	0.505
**Composite dimensions**					
Problem-focused coping	0–18	10.96 ± 3.68	1.83	0.811	
Emotion-focused coping	0–30	14.59 ± 5.02	1.46	0.758	
Dysfunctional coping	0–36	12.98 ± 6.40	1.08	0.836	

Note: SD = standard deviation. Standardized mean = total score divided by the number of items, expressed on the original 0–3 response metric. Inter-item correlations are reported for the original two-item Brief-COPE subscales because Cronbach’s α is sensitive to the number of items. For the multi-item composite dimensions, Cronbach’s α is reported.

**Table 9:** Associations between the raw score of the Meister Questionnaire and coping strategies assessed using the Brief-COPE Inventory.

Original Brief-COPE Subscales	Spearman’s ρ	Unadjusted *p*-Value	Holm-Adjusted *p*-Value
Active coping	−0.148	<0.001	0.003
Emotional support	−0.294	<0.001	<0.001
Instrumental support	−0.169	<0.001	<0.001
Positive reframing	−0.043	0.329	0.467
Planning	−0.233	<0.001	<0.001
Venting	0.154	<0.001	0.002
Humor	0.229	<0.001	<0.001
Acceptance	−0.053	0.230	0.467
Self-blame	0.245	<0.001	<0.001
Religion	0.124	0.006	0.015
Self-distraction	0.160	<0.001	0.002
Substance use	0.349	<0.001	<0.001
Denial	0.255	<0.001	<0.001
Behavioral disengagement	0.300	<0.001	<0.001
**Composite dimensions**			
Problem-focused coping	−0.224	<0.001	<0.001
Emotion-focused coping	−0.018	0.680	0.680
Dysfunctional coping	0.356	<0.001	<0.001

Note: Holm correction was applied separately to the 14 original Brief-COPE subscales and the three composite dimensions. Statistical significance was determined based on Holm-adjusted *p*-values, with *p* < 0.05 considered statistically significant.

**Table 10:** Multivariable linear regression analyses of associations between coping strategies and individual Meister Questionnaire raw scores.

Predictor	B	SE	β	95% CI for B	*p*-Value	Overall *p*-Value
**Panel A. Primary strategy-level model (14 original Brief-COPE subscales)**						
Self-distraction	0.740	0.290	0.130	0.170 to 1.309	0.011	
Active coping	0.074	0.308	0.012	−0.531 to 0.680	0.810	
Denial	0.282	0.305	0.048	−0.318 to 0.882	0.356	
Substance use	1.006	0.278	0.196	0.461 to 1.552	<0.001	
Emotional support	−1.728	0.339	−0.313	−2.395 to −1.061	<0.001	
Behavioral disengagement	0.417	0.311	0.072	−0.194 to 1.027	0.181	
Venting	0.612	0.322	0.103	−0.021 to 1.245	0.058	
Instrumental support	0.294	0.347	0.054	−0.387 to 0.975	0.397	
Self-blame	0.015	0.271	0.003	−0.519 to 0.548	0.957	
Positive reframing	0.051	0.308	0.009	−0.555 to 0.657	0.868	
Planning	−0.534	0.333	−0.092	−1.189 to 0.121	0.110	
Acceptance	−0.400	0.314	−0.069	−1.018 to 0.218	0.204	
Religion	0.510	0.211	0.106	0.096 to 0.924	0.016	
Humor	0.148	0.297	0.026	−0.434 to 0.731	0.617	
**Sociodemographic and occupational covariates**						
Sex: Male (vs. Female)	−1.260	1.210	−0.042	−3.637 to 1.117	0.298	
Age: 31–40 (vs. <30)	1.594	1.074	0.076	−0.516 to 3.704	0.138	0.058
Age: 41–50 (vs. <30)	−1.972	1.318	−0.085	−4.562 to 0.618	0.135	
Age: ≥51 (vs. <30)	−1.064	1.973	−0.039	−4.941 to 2.813	0.590	
Education: Bachelor’s (vs. Secondary)	−1.465	0.977	−0.086	−3.385 to 0.455	0.135	0.224
Education: Master’s/Doctoral (vs. Secondary)	−0.438	1.156	−0.024	−2.709 to 1.833	0.705	
Practice: 5–10 (vs. <5)	−1.534	1.332	−0.055	−4.152 to 1.084	0.250	0.159
Practice: 11–20 (vs. <5)	0.585	1.462	0.020	−2.289 to 3.458	0.690	
Practice: 21–30 (vs. <5)	2.856	1.671	0.108	−0.427 to 6.139	0.088	
Practice: ≥31 (vs. <5)	0.244	2.291	0.007	−4.258 to 4.746	0.915	
Shift: Multi-shift (vs. Single-shift)	0.806	0.747	0.047	−0.662 to 2.273	0.281	
Care: Outpatient (vs. Inpatient)	−0.794	1.028	−0.031	−2.814 to 1.226	0.440	
**Model A fit: *R*^2^ = 0.283; adjusted *R*^2^ = 0.245; *F*(26, 487) = 7.40; *p* < 0.001.**						
**Panel B. Complementary composite-level model**						
Problem-focused coping	−0.553	0.118	−0.239	−0.785 to −0.322	<0.001	
Emotion-focused coping	−0.064	0.097	−0.037	−0.254 to 0.127	0.513	
Dysfunctional coping	0.569	0.062	0.427	0.447 to 0.692	<0.001	
**Sociodemographic and occupational covariates**						
Sex: Male (vs. Female)	−0.272	1.217	−0.009	−2.664 to 2.120	0.824	
Age: 31–40 (vs. <30)	1.709	1.067	0.081	−0.388 to 3.806	0.110	0.127
Age: 41–50 (vs. <30)	−1.231	1.328	−0.053	−3.840 to 1.378	0.354	
Age: ≥51 (vs. <30)	0.125	1.985	0.005	−3.775 to 4.024	0.950	
Education: Bachelor’s (vs. Secondary)	−1.710	0.989	−0.100	−3.653 to 0.233	0.084	0.159
Education: Master’s/Doctoral (vs. Secondary)	−0.660	1.169	−0.036	−2.956 to 1.636	0.573	
Practice: 5–10 (vs. <5)	−0.689	1.338	−0.025	−3.317 to 1.940	0.607	0.294
Practice: 11–20 (vs. <5)	0.577	1.475	0.020	−2.322 to 3.475	0.696	
Practice: 21–30 (vs. <5)	2.323	1.679	0.087	−0.976 to 5.621	0.167	
Practice: ≥31 (vs. <5)	−1.029	2.311	−0.029	−5.570 to 3.512	0.656	
Shift: Multi-shift (vs. Single-shift)	0.749	0.756	0.043	−0.736 to 2.235	0.322	
Care: Outpatient (vs. Inpatient)	−0.316	1.039	−0.012	−2.357 to 1.726	0.761	
**Model B fit: *R*^2^ = 0.226; adjusted *R*^2^ = 0.203; *F*(15, 498) = 9.69; *p* < 0.001.**						

## Data Availability

The data presented in this study are not publicly available because they are part of the ongoing Nursing Workload research project funded by the Cultural and Educational Grant Agency of the Ministry of Education, Research, Development and Youth of the Slovak Republic (KEGA), grant No. 011KU-4/2024, and will be used in future analyses. Data may be made available by the corresponding author upon reasonable request, subject to ethical considerations.

## Abbreviations

The following abbreviations are used in this manuscript:

ANOVA	Analysis of Variance
Brief-COPE	Brief Coping Orientation to Problems Experienced Inventory
CI	Confidence Interval
HC3	Heteroskedasticity-Consistent Standard Errors
HS	Overall Raw Score (according to the Meister Questionnaire methodology)
KEGA	Cultural and Educational Grant Agency
Me	Median
NASA-TLX	National Aeronautics and Space Administration Task Load Index
NIC	Nursing Interventions Classification
OECD	Organisation for Economic Co-operation and Development
SE	Standard Error
SD	Standard Deviation
VIF	Variance Inflation Factor
